# Soil metagenomic analysis on changes of functional genes and microorganisms involved in nitrogen-cycle processes of acidified tea soils

**DOI:** 10.3389/fpls.2022.998178

**Published:** 2022-10-14

**Authors:** Shunxian Lin, Zhijun Liu, Yuchao Wang, Jiayu Li, Gege Wang, Jianghua Ye, Haibin Wang, Haibin He

**Affiliations:** ^1^ Key Laboratory of Agroecological Processing and Safety Monitoring of Fujian Province, Fujian Agriculture and Forestry University, Fuzhou, China; ^2^ College of Tea and Food Science, Wuyi University, Wuyishan, China; ^3^ College of Life Sciences, Longyan University, Longyan, China

**Keywords:** tea (Camellia sinensis), soil acidification, nitrogen forms, nitrogen functional genes, microbial composition

## Abstract

Nitrogen (N) is the first essential nutrient for tea growth. However, the effect of soil acidification on soil N cycle and N forms in tea plantation are unclear. In this study, the nitrogen contents, soil enzyme activity and N mineralization rate in acidified soil of tea plantation were measured. Moreover, the effects of soil acidification on N cycling functional genes and functional microorganisms were explored by soil metagenomics. The results showed that the NH_4_^+^-N, available N and net N mineralization rate in the acidified tea soil decreased significantly, while the NO_3_^-^-N content increased significantly. The activities of sucrase, protease, catalase and polyphenol oxidase in the acidified tea soil decreased significantly. The abundance of genes related to ammonification, dissimilatory N reduction, nitrification and denitrification pathway in the acidified tea soil increased significantly, but the abundance of functional genes related to glutamate synthesis and assimilatory N reduction pathway were opposite. In addition, the abundance of Proteobacteria, Actinobacteria, Chloroflexi, Nitrospirae, *Actinomadura*, *Nitrospira* etc. microorganisms related to nitrification, denitrification and pathogenic effect increased significantly in the acidified tea soil. The correlation results showed that soil pH and N forms were correlated with soil enzyme activity, N cycling function genes and microbial changes. In conclusion, soil acidification results in significant changes in enzyme activity, gene abundance and microorganism involved in various N cycle processes in acidified tea soil, which leads to imbalance of soil N form ratio and is not conducive to N transformation and absorption of tea trees.

## Introduction

Tea (*camellia sinensis*) is an important economic plant in tropical and subtropical countries. Tea is a perennial leaf-harvested crop that grows best in acidic soil with pH value of- 4.5-5.5 (Yan et al., 2020). However, soil acidification is major problem in tea plantation. Generally, excessive N fertilization is considered the major driver of soil acidification in tea plantations (Huang et al., 2015a; Yan et al., 2020; Lin et al., 2022). Specifically, the accelerated soil acidification from fertilization is directly caused by the production of protons *via* the nitrification process after application of ammonium N fertilization (Barak et al., 1997; Zhou et al., 2014). In addition, the nitrate nitrogen (NO_3_^–^–N) produced by nitrification was washed out of the soil under heavy rainfall and took away a large number of base ions, resulting in the decrease of the acid neutralization ability of the soil (Yang et al., 2018b). Moreover, not all of the large amount of fertilizer applied can be absorbed and utilized by plants, and the loss of N from agroecosystems has led to pollution of groundwater, nitrous oxide (N_2_O) emissions, and other environmental problems (Sun et al., 2021).

Nitrogen is an essential element for all plants to grow and reproduce. NH_4_^+^-N and NO_3_^–^–N are the main types of available N, but they are limited in the natural environment. Only after N mineralization process, organic N can be converted into effective N (NO_3_^-^, NH_4_^+^), which can be absorbed and utilized by plants (Robertson et al., 1999). Compared with NO_3_^-^, NH_4_^+^ could significantly improve the photosynthetic efficiency and quality of tea trees (Ruan et al., 2008). N transformation in soil refers to the process of changing the form or state of N-containing substances in soil by physical chemistry and biochemistry, mainly includes N mineralization, N fixation, nitrification and denitrification etc. (Kuypers et al., 2018). Transformation of soil N forms is carried out by microbiome containing specific functional genes (Zhang et al., 2019). There are many studies focused on soil N transformation, but mainly focuses on the effects of different fertilization management on soil N transformation (Saggar et al., 2013). For example, for acidic soils, long-term application of chemical N fertilizer resulted in increased activity and abundance of Ammonia oxidizing archaea (AOA) (Leininger et al., 2006). Sun et al. (2020) reported that the application of biological fertilizer reduced the abundance of bacterial *amoA* gene in soil, but enhanced the soil denitrification, reduced the accumulation of NO_3_^–^–N, thus greatly reduced N leaching loss in runoff. The addition of Oxytetracycline inhibited nitrifying bacteria reduce the conversion of NH_4_^+^-N to NO_3_^–^–N in composting process (Zhang et al., 2021). The use efficiency of soil N can be improved by applying different fertilizers. Although there are a lot of research on soil N genes, most of these studies used qRT-PCR methods, which often lead to biased results and focus on a single gene involved in the N cycle or single N process-related genes. N cycle is a network of interconnected processes. Such an approach lacks information on the complete N-cycle metabolism process, which can lead to the loss of important information.

Soil pH value is an important factor influencing soil N-related transformation and microbial communities (Wang et al., 2017). Previous studies have demonstrated that soil acidification will lead to the loss of soil base ions, the decrease of directly available NH_4_^+^-N and NO_3_^–^–N in soil, the intensification of soil acidification, and then decrease of N conversion efficiency in soil (Yamamoto et al., 2014; Huang et al., 2015b). Soil enzymes play an important role in material cycle and energy flow in soil ecosystem, which regulate the conversion and circulation of soil nutrients. Meanwhile, the study of the composition of soil microbial community is conducive to an in-depth understanding of the soil internal ecological processes. Soil acidification can change the diversity of soil microbial community structure, such as increasing the relative abundance of acidifying bacteria bacillus, and decreasing the number of Proteobacteria, Bacteroides and Actinomycetes (Xu et al., 2016; Goswami et al., 2017). Planctomycetes, Acidobacteria, Nitrospirae etc. Microorganisms are closely related to soil nitrogen cycling (Zhou et al., 2018; Spieck et al., 2020; Hu et al., 2021). Tea plants have a great demand for N as the buds and leaves are the main subjects for tea yield. Then, can soil acidification influence the content of soil N? And the response of the functional genes and microorganisms involved in the N cycle to soil acidification in tea plantation and their relationship with N forms are unclear.

Here, soil N contents, N mineralization rates and soil enzyme activity with different soil pH values were measured. Furthermore, metagenomic methods were used to comprehensively analyze the differences of various functional genes in soil N-cycling process and microbial communities of tea plantation with different pH values. The objective of this study is 1) to analyze the change of N contents and soil enzyme activity in tea soil after acidification; 2) to assess the response of the functional genes involved in the N cycle and functional microorganisms to soil acidification and their relationship with N forms, and 3) to analyze the specific genes involved in different N processes with different acidity of tea soils.

## Materials and methods

### Sample collection and site description

The experiment site was located in Nanjing County, Fujian Province, China (24°37′04″N, 117°02′80″E), elevation 590-582 m above sea level, The site is a typical monsoonal with a mean annual temperature of 21.40°C. The minimum and maximum mean monthly temperatures are -0.5°C and 38.9°C in January and July, respectively, with a mean annual rainfall of 1821 mm. The site has a haplic arenosol soil (FAO classification) and with a clay loam texture. A 750 kg ha^−1^ of compound fertilizer (17% N, 17% P, and 17% K) and 600 kg ha^−1^ of urea were added to the soil in mid-February of every year. In late-May and July, a compound fertilizer and urea were added to the soil surface at the rate of 750 and 300 kg ha^-1^ each time. The pruning of upper leaves and buds were reserved in tea plantation for surface mulch.

In August 2020, based on the results of previous studies (Lin et al., 2022), tea plantations in the same region with soil pH ranges of 3.5-4.0 (AT) and 5.0-5.5 (ST) were selected as research sites. Each tea plantation plot area is no less than 40 m^2^ (4 m × 10 m). Then soil samples were randomly collected from three plots in each site. The rhizosphere soil of tea trees was taken from each experimental plot by a five-point sampling method. Briefly, the surface deciduous leaves of the selected trees were removed. The 500 g soils were collected from the tea tree rhizosphere at a radius of 10-15 cm and depth of 10-20 cm, then mixed thoroughly to form one composite sample. Different soil samples were immediately placed into plastic bags, to minimize exposure to O_2_, and prior to analyses. The fresh soil was sieved in a 2-mm-mesh sieve and divided into three subsamples. One portion dried in air naturally was used to measure chemical properties. One portion was stored at 4°C for enzyme activity determination. And the remainder was immediately frozen at -80°C for DNA analysis.

### Chemical properties determining

The pH of soil was determined with a glass electrode (PB-10; Sartorius, Shanghai, China) with a soil to water ratio of 1:2.5. The contents of NO_3_^–^–N and NH_4_^+^-N in soil samples were determined according to the method of Lu (1999). In brief, 5 g soil sample was used to extract and the NO_3_^–^–N content was determined using dual-wavelength spectrophotometry at 210 nm and 275 nm. The 10 g of soil sample was used to extract and the NH_4_^+^-N content was measured using indigophenol blue colorimetric method at 625 nm. The sum of NO_3_^–^–N and NH_4_^+^-N contents was the available N (AN) content. The soil net N mineralization rate (Mit_r_) was determined by measuring the change of available N (NH_4_^+^-N and NO_3_^–^–N) in the soil samples. In brief, 10 g of tea soils were incubated in a 100 mL culture flask and cultured in the dark at 25°C for 7 d (Li et al., 2020). Soil mineral N (NH_4_^+^-N and NO_3_^–^–N) was determined by colorimetric method at the beginning and the end of the culture, then the N mineralization rate was analyzed. All soil properties were determined in triplicate.

### Determination of soil enzyme activity

The soil enzyme activity was analyzed by the reference (Guan et al., 1986). Briefly, sucrase activity (SC) was determined using 3,5-dinitrosalicylic acid colorimetry; urease activity (UR) was determined using sodium phenol colorimetry; protease activity (PR) was determined using Folin colorimetric method; catalase (CAT) activity was determined by potassium permanganate titration; polyphenol oxidase activity (PPO) was determined by purple gallic acid colorimetric method; and phosphomonoesterase activity (PMase) was determined by *p*-nitrophenylphosphate disodium colorimetric method. All soil properties were determined in triplicate.

### DNA extraction and metagenomics sequencing

Soil total DNA extraction were using Bio-Fast Soil Genomic DNA Extraction kit (BioFlux, Hangzhou, China), according to the manufacturer’s instructions with 0.5 g. The integrity of DNA was checked by 1% agarose gels. and the concentration was measured using a NanoDrop ND-1000 spectrophotometer (Thermo Fisher Scientific, Waltham, MA, USA). The pure DNA in the extraction solution was processed with the Illumina TruSeq Nano DNA LT Library Preparation Kit to construct metagenome shotgun sequencing libraries with insert sizes of 350 bp. Each library was sequenced by using the Illumina HiSeq X-ten platform (Illumina, USA) with the PE150 strategy.

Preprocessing the Raw Data obtained from the Illumina HiSeq sequencing platform were preprocessed by Readfq to obtain the Clean Data for subsequent analysis. After obtaining quality-filtered reads, they were *de novo* assembled and use MEGAHIT (v1.0.4-beta) to construct the metagenome for each sample. The Scaftigs (> 300 bp) assembled from both single and mixed are all predicted the ORF by MetaGeneMark (Zhu et al., 2010), then were clustered by CD-HIT (V4.5.8) to redundancy and obtain non-redundant gene catalogue. Gene abundance in each sample was estimated by using soap. coverage (http://soap.genomics.org.cn/) based on the number of aligned reads. And then using BLASTP (BLAST Version 2.2.28 + 5) to compare the gene set and NR database (e-value ≤ 1e^−5^), N cycle microbial species annotation was obtained through the gene taxonomic information database corresponding to the NR database, and then species abundance was calculated by summing the abundances of genes corresponding to the species (Wang et al., 2020).

### Statistical analysis

The changes in soil chemical properties, soil enzyme activity and gene abundance were tested for significant differences among tea soils with one-way ANOVA using the SPSS 20.0 (SPSS Inc., Chicago, IL, USA) followed by the least significant difference (LSD) at a 5% level of probability. All experimental data were showed as the mean ± standard error. Graphs were prepared using origin (OriginPro 9.0) unless otherwise indicated. Pearson correlation analysis was used to examine the relationships among the environmental factors, soil enzyme activity and abundance of functional genes for each N transformation, using the Corrplot program package in R software. Redundancy analysis (RDA) was carried out to examine the relationships between environmental factors and microbial abundance at the phylum and genus level, using the vegan package in R software.

## Results

### Soil physicochemical properties

The soil properties varied significantly between the two tea soils (**Table 1**). The pH of tea soil with AT and ST were 3.75 and 5.26, respectively. With the increase of soil pH value, the contents of NO_3_^–^–N were 41.10 and 29.87 mg·kg^-1^, respectively. After acidification, the NO_3_^–^–N content of tea soil significantly increased (*P* < 0.05) by 11.23 mg·kg^-1^. In contrast, relative to ST, the content of NH_4_^+^-N in AT was significantly decreased (*P* < 0.05) by 15.91 mg·kg^-1^, and corresponding decrease for available N was 4.67 mg·kg^-1^. With the increase of soil pH value, the rate of net N mineralization were 2.29 and 5.70 mg·kg^-1^ d^-1^, respectively. The net N mineralization rate of ST were about 2.5 times as much as that of AT (acidified soil).

**Table 1:** physiochemical properties of tea soils with different pH values.

Physicochemical property	pH	NH_4_^+^-N (mg kg^-1^)	NO_3_^–^–N (mg kg^-1^)	AN (mg kg^-1^)	OM (mg kg^-1^)	Mit_r_ (mg kg^-1^ d^-1^)
AT	3.75 ± 0.02b	4.91 ± 0.27b	41.10 ± 0.06a	46.02 ± 0.22b	36.28 ± 0.18a	2.29 ± 0.02b
ST	5.26 ± 0.02a	20.82 ± 0.26a	29.87 ± 0.07b	50.69 ± 0.22a	33.46 ± 0.16b	5.70 ± 0.01a

AT, tea soil with a pH of 3.75; ST, tea soil with a pH of 5.26. NH_4_^+^-N, ammonium nitrogen content; NO_3_^–^–N, nitrate nitrogen content; AN, available nitrogen content; OM, organic matter content; Mit_r_, net nitrogen mineralization rate. Data in the table are mean ± SE. Different lowercases represent significant differences at P < 0.05 level.

### Soils enzymatic activity

**Figure 1:**
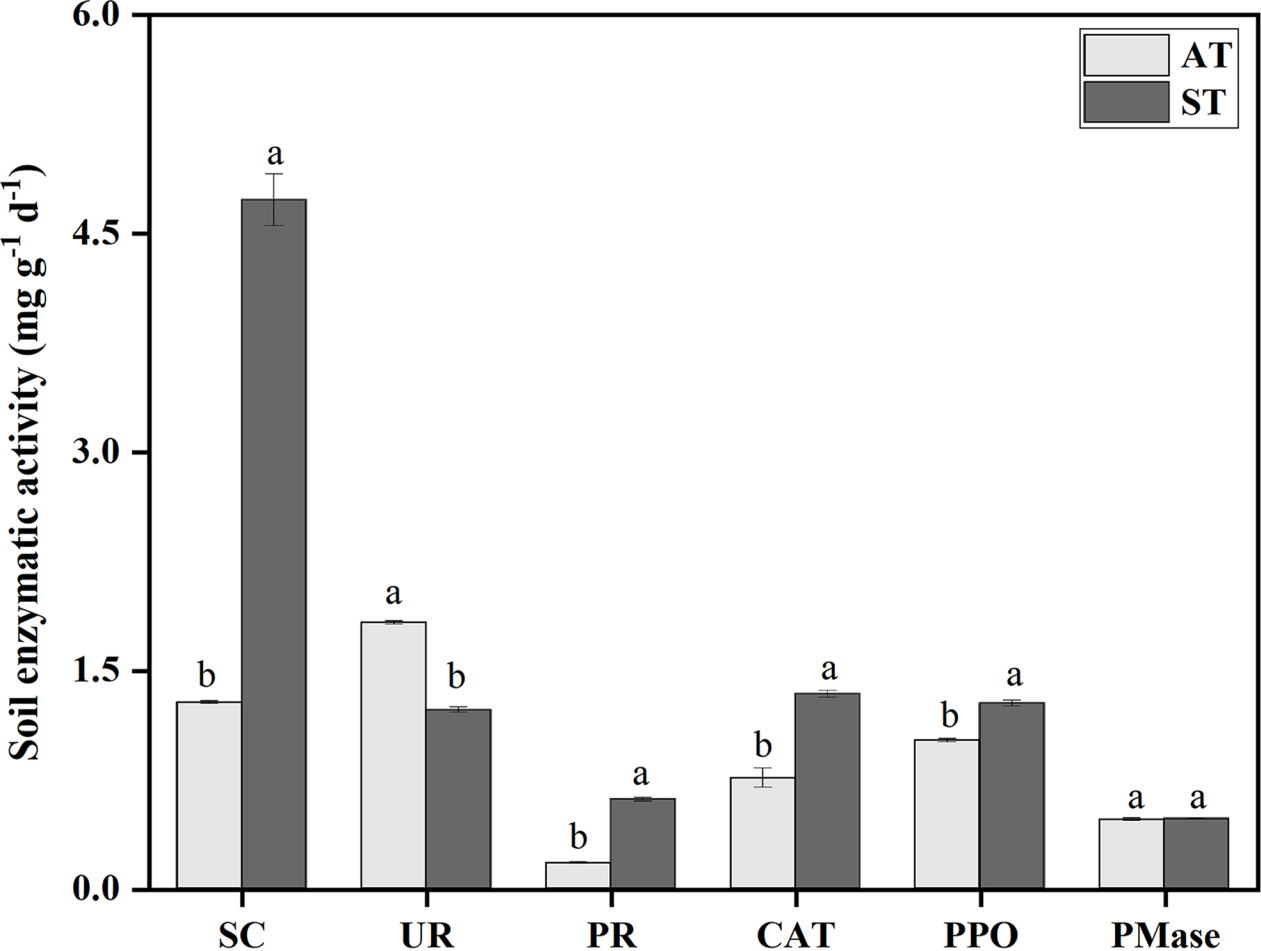
Soil enzyme activities in tea soil with different pH values. SC, sucrase; UR, urease; PR, protease; CAT, catalase; PPO, polyhphenol oxidase; PMase, phosphomonoesterase. AT, tea soil with a pH of 3.75; ST, tea soil with a pH of 5.26. Bars indicate standard error of the mean (n = 3). Different lowercases represent significant differences at *P* < 0.05 level.

There were significant differences (*P* < 0.05) in soil enzyme activities with different pH values (**Figure 1**). The activity of sucrase was significantly higher than that of other enzymes. Compared with ST, the activities of sucrase, protease, catalase and polyhphenol oxidase decreased by 3.45, 0.44, 0.58 and 0.25 mg g^-1^ h^-1^ under AT, respectively. And urease activity of AT was significantly higher than that of ST (*P* < 0.05). Taken together, the activities of sucrase, protease, catalase and polyphenol oxidase in tea soil decreased significantly, and urease activity increased significantly after soil acidification, but there was no significant difference in phosphomonoesterase activity.

### Functional N transformation gene shifts

A total of 76720.18 Mbp of raw reads was obtained from 6 libraries after the Illumina sequencing. After filtering, 76532.51 Mbp of clean reads were identified, and the percentage of clean reads relative to raw reads in each library was above 99.76% (**Supplementary Table S1**), indicating that the results could comprehensively and truly reflect the composition of soil microbial community. We further summarized and analyzed the frequency and abundance of functional genes involved in the complete N-cycle metabolic process.

**Figure 2:**
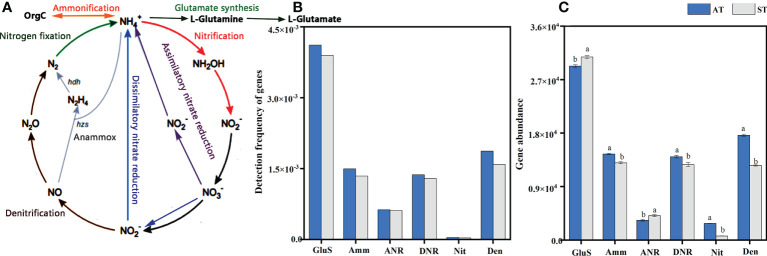
An overall schema illustrating the N-cycle processes **(A)**, detection frequency **(B)** and abundance **(C)** of functional genes for each N-cycle with different acidity of tea soils. AT, tea soil with a pH of 3.75; ST, tea soil with a pH of 5.26. GluS, glutamate synthesis; Amm, ammonification; ANR, assimilatory nitrogen reduction; DNR, dissimilatory nitrogen reduction; Nit, nitrification; Den, denitrification. Bars indicate standard error of the mean (n = 3). Different lowercases represent significant differences at *P* < 0.05 level.

A total of 11049 functional gene probes were detected (**Supplementary Table S2**), which included 28 genes and 6 pathways of N-cycle processes including glutamate synthesis, ammonification, assimilatory N reduction, dissimilatory N reduction, nitrification and denitrification. The processes of N-cycle are shown in **Figure 2A**. The frequency of genes involved in glutamate synthesis was the highest, with 4114 and 3895 functional genes detected by AT and ST, respectively. In contrast, only 41 and 33 functional genes were detected by nitrification (**Figure 2B**). However, genes involved in N fixation and anammox were not captured in this study (**Figure 2B**).

Overall, the total abundance of genes involved in N pathways in AT was significantly higher (*P* < 0.05) than that in ST (**Supplementary Figure S1**). With the increase of soil pH, the abundances of genes involved in N pathways were 8.05 × 10^4^ and 7.22 × 10^4^, respectively (**Supplementary Figure S1B**). For abundance of functional genes for each N-cycle processes, the abundance of functional genes involved in glutamate synthesis pathway was the highest and significantly higher (*P* < 0.05) than those genes in other N-cycle processes (**Figure 2C**). After soil acidification, the abundance of genes related to ammonification, dissimilatory N reduction, nitrification and denitrification pathway in tea soils increased significantly (*P* < 0.05). And the abundance of genes involved in glutamate synthesis and assimilatory N reduction pathways decreased significantly (*P* < 0.05) under soil acidification (**Figure 2C**).

**Figure 3:**
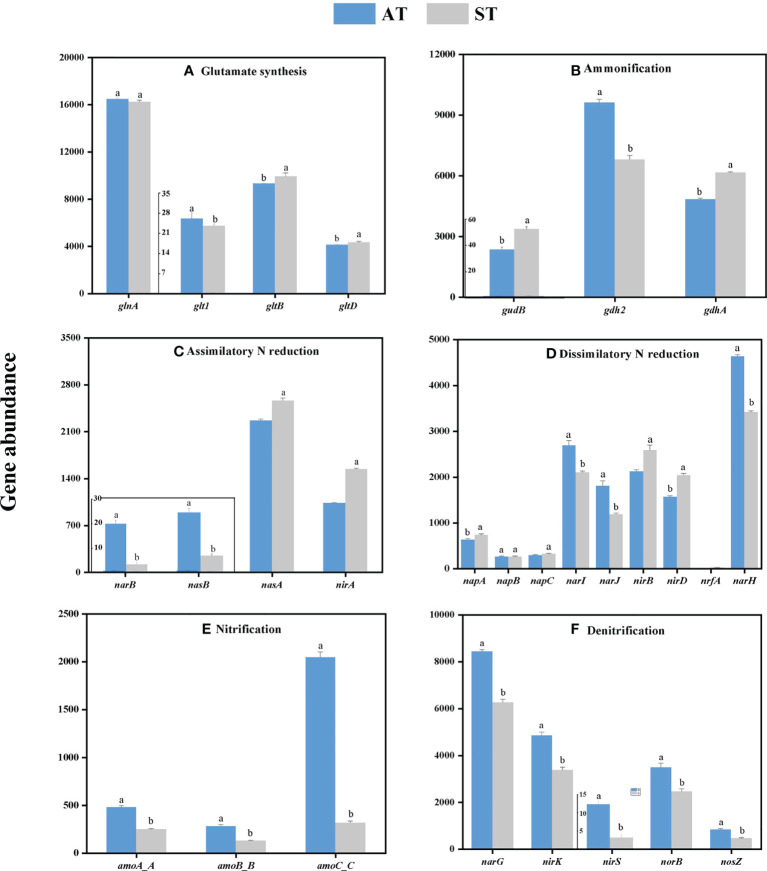
The abundance of individual genes involved in each N-cycle processes **(A–F)** with different acidity of tea soils. AT, tea soil with a pH of 3.75; ST, tea soil with a pH of 5.26. Bars indicate standard error of the mean (n = 3). Different lowercases represent significant differences at *P* < 0.05 level.

From the perspective of individual genes, soil acidification significantly changed the individual gene abundance in tea soils (**Figure 3**). Overall, soil acidification significantly increased (*P* < 0.05) the abundances of *gdh2* involved in ammonification (**Figure 3B**), *narB* and *nasB* involved in denitrification (**Figure 3C**), *narI*, *narJ* and *narH* involved in dissimilatory N reduction (**Figure 3D**), and all individual genes involved in nitrification (*amoA_*ABC) (**Figure 3E**) and denitrification (*narG*, *nirK*, *nirS*, *norB* and *nosZ*) (**Figure 3F**). In contrast, the abundances of *gltB* and *gltD* involved in glutamate synthesis (**Figure 3A**), *gudB* and *gdhA* involved in ammonification (**Figure 3B**), *nasA* and *nirA* involved in assimilatory N reduction (**Figure 3C**), and *napA*, *napC*, *nirB*, *nirD* and *nrfA* involved in dissimilatory N reduction (**Figure 3D**) decreased significantly (*P* < 0.05). The abundances of different individual genes involved in the same pathway were not showed a consistent trend with the change of soil pH value.

### Microorganisms involved in the nitrogen cycle

**Figure 4:**
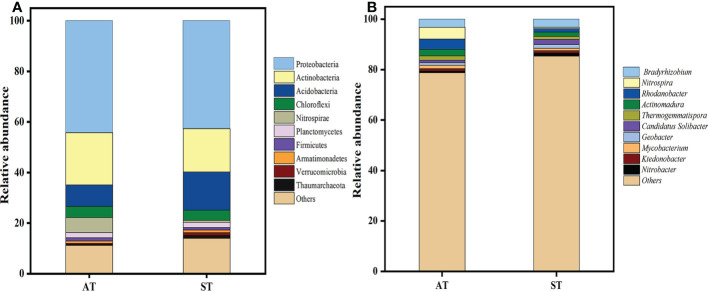
Distribution of nitrogen-related total microbial top 10 phyla **(A)** and genus **(B)** with different pH values of tea soils. AT, tea soil with a pH of 3.75; ST, tea soil with a pH of 5.26.

The PCA plots indicated that significant differences occurred in soil N-cycling microbial communities with different pH values. The N-cycling microbial were well separated in the first component (PC1) (**Supplementary Figure S2**), with the three replicates displaying close clustering. At the phylum level, a total of 26 phyla was detected by metagenomic sequencing from two soil samples (**Supplementary Figure S3**). The top 10 soil microbial communities at the phylum level, accounting for 87.41% of the total soil microorganisms were further selected (**Figure 4A**). The relative abundances of Proteobacteria, Actinobacteria, Chloroflexi, Nitrospirae and Firmicutes in AT were significantly higher (*P* < 0.05) than those in ST, while the abundance of Acidobacteria, Armatimonadetes, Verrucomicrobia and Thaumarchaeota were the opposite. Taken together, with the increase of soil pH, the total abundance of top 10 species of soil N-cycling microbial communities at the phylum level accounted for 88.84 and 86.00% of the total, respectively (**Supplementary Table S3**).

A total of 326 microorganisms genera was obtained at the genus level. As shown in **Figure 4B**, there were significant differences in soil microbial abundance at genus level in tea soils with different pH values. Compared with ST, the relative abundance of *Nitrospira, Rhodanobacter*, *Actinomadura*, *Thermogemmatispora* and *Mycobacterium* in AT increased significantly (*P* < 0.05), while that of *Candidatus Solibacter*, *Geobacter*, *Ktedonobacter* and *Nitrobacter* decreased significantly (*P* < 0.05). There was no significant difference in *Bradyrhizobium* between AT and ST.

### Correlation between soil microorganism, soil enzyme activity and environmental parameters

**Figure 5:**
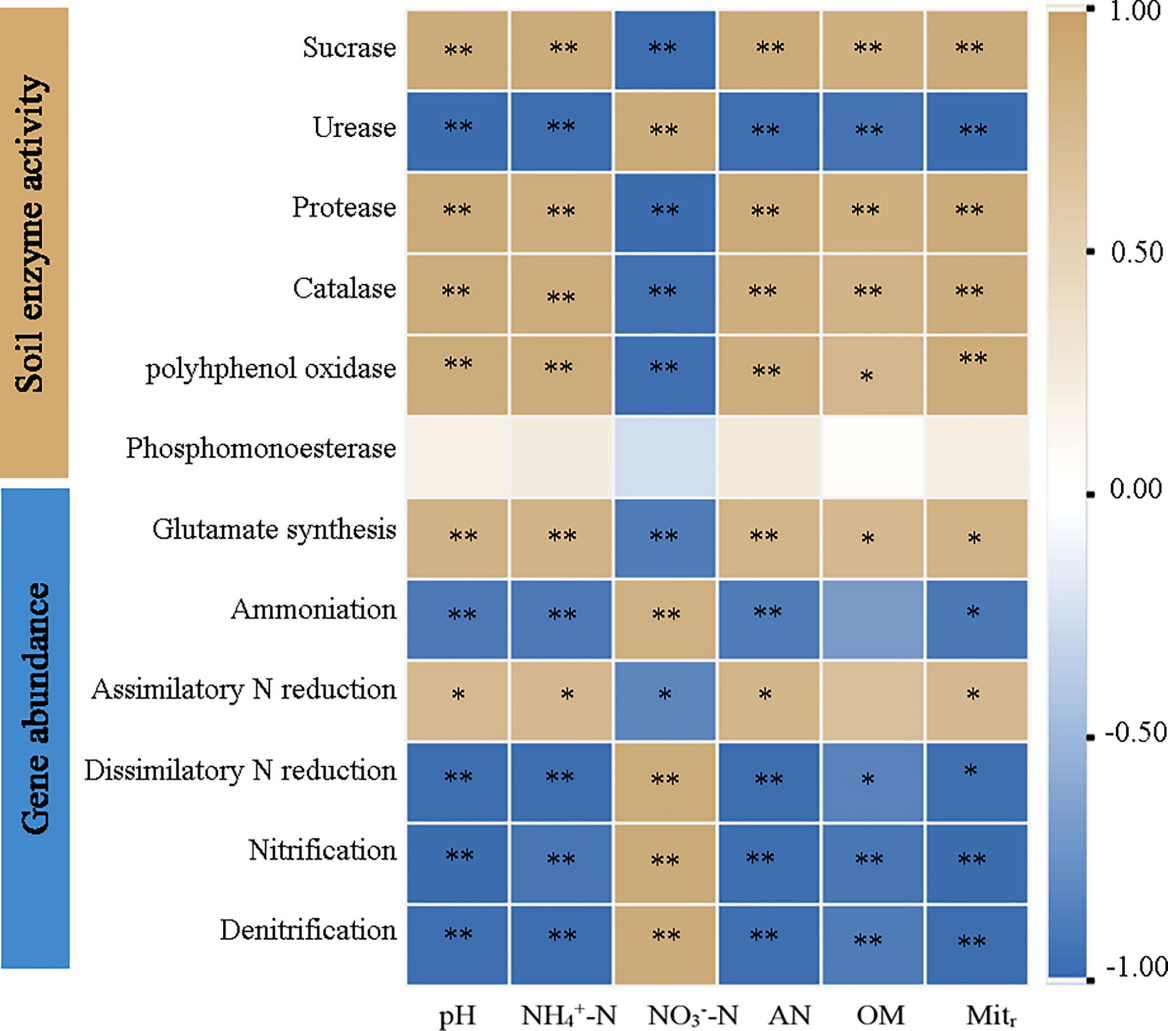
Heatmap of pearson correlation analysis of soil physicochemical properties, enzyme activity and functional gene abundance in tea soil with different pH values. NH_4_^+^-N, ammonium nitrogen content; NO_3_^–^–N, nitrate nitrogen content; AN, available nitrogen content; OM, organic matter content; Mit_r_, net nitrogen mineralization rate. * and **, significant at *P* ≤ 0.05 and *P* ≤ 0.01.

Pearson correlation analysis identified significant correlations (*P* < 0.05) between physicochemical properties and N-related gene abundances across AT and ST (**Figure 5**). The pH value, NH_4_^+^-N, available N and net N mineralization rate were significantly positively correlated (*P* < 0.05) with activities of sucrase, protease, catalase, and polyhphenol oxidase, and the gene abundances involved in glutamate synthesis and assimilatory N reduction, whereas they were significantly negatively related (*P* < 0.05) to urease activity and the gene abundances involved in ammonification, dissimilatory N reduction, nitrification and denitrification. The correlation between NO_3_^–^–N content and other indexes was contrary to that of NH_4_^+^-N and available N (*P* < 0.05).

**Figure 6:**
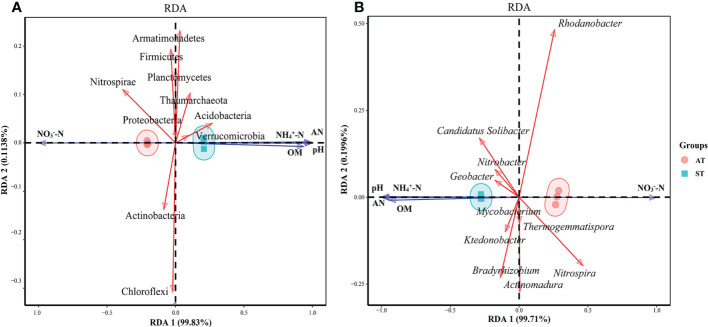
Canonical redundancy analysis (RDA) of soil physicochemical factor and dominant phyla **(A)** and genera **(B)** microorganism in three soil sample. AT, tea soil with a pH of 3.75; ST, tea soil with a pH of 5.26.

RDA was performed using the top 10 species of microbial taxa at the phylum level and soil physicochemical properties (**Figure 6**). The results showed that axes 1 and 2 together described 99.94% of the variation in microorganisms at the phylum level and 99.91% of microorganisms at the genus level, indicating a significant correlation between soil physicochemical factors and microbes (*P* < 0.05) (**Figure 6**). As for the microorganisms at the phylum level (**Figure 6A**), soil pH, NH_4_^+^-N and available N content and net N mineralization rate were significantly positively correlated (*P* < 0.05) with the abundances of Acidobacteria, Armatimonadetes, Verrucomicrobia and Thaumarchaeota, and significantly negatively correlated (*P* < 0.05) with the abundance of Proteobacteria, Actinobacteria, Chloroflexi, Nitrospirae, Planctomycetes and Firmicutes. And the correlation between NO_3_^–^–N content and other indexes were contrary to that of NH_4_^+^-N content (*P* < 0.05).

As for the microorganisms at the genus level (**Figure 6B**), soil pH, NH_4_^+^-N, available N content and net N mineralization rate were positively correlated (*P* < 0.05) with the abundances of *Candidatus Solibacter*, *Geobacter* and *Nitrobacter* and negatively correlated (*P* < 0.05) with the abundances of Bradyrhizobium, *Nitrospira*, *Rhodanobacter*, *Actinomadura*, *Thermogemmatispora* and *Mycobacterium*. And the correlation between NO_3_^–^–N content and other indexes were contrary to that of NH_4_^+^-N and available N (*P* < 0.05).

## Discussion

### Soil nitrogen form and transformation

Nitrogen involved in a lot of biological processes in terms of different level and forms (NH_4_^+^ and/or NO_3_^−^) (Huang et al., 2018). Tea plants prefer NH_4_^+^ rather than NO_3_^−^, and NH_4_^+^ can promote the growth of tea plants more effectively (Huang et al., 2018; Wang et al., 2021). In our study, the contents of NH_4_^+^-N and available N in acidified soil decreased significantly, but the content of NO_3_^−^-N was higher than that in soil suitable for tea tree growth (**Table 1**). These results indicated that soil pH affect the N forms of tea soils, and soil acidification lead to the decrease of available N for tea plant. By further analyzing the soil N transformation capacity, we found that the rate of net N mineralization in acidified soil decreased significantly (**Table 1**). Consistent with our findings, previous researchers reported that the rate of N mineralization increases as the increase of pH values of 4-8 (Jiang et al., 2015). Mineralization results in increasing the content of available N in plant soil (Robertson and Groffman, 2007), which indicated that acidification would reduce soil mineralization capacity, thus lead to a decline in soil N supply capacity and a decrease of N absorption and utilization capacity of tea trees.

### Soil enzyme activity

Soil enzyme activity is an indicator of soil quality, which reflect the direction and intensity of biochemical processes and nutrient cycles in soil (Xu et al., 2021). The activities of N-cycling enzymes such as urease, catalase and protease existed significantly differences under different N fertilizer treatments (He et al., 2021; Li et al., 2021). Our study found that the activities of sucrase, protease, catalase and polyphenol oxidase in acidified tea soil decreased significantly (**Figure 2**). Soil enzymes such as sucrase and catalase were closely related to soil organic matter accumulation and saprophytic degree, where their debris and subsequent nutrients release into the soil is made available to plants (Dick, 1994; Madejón et al., 2007). Protease can hydrolyze various protein and peptide compounds into amino acids, which is one of the N sources of higher plants (Nie et al., 2020). Polyphenol oxidase has a defense effect against soil pathogens (Mayer, 2006). It could be seen that acidification of tea soil has a negative effect on soil carbon and N conversion, N supply capacity, disease resistance and soil humus content.

### Functional N transformation gene shifts

The changes of soil functional gene abundance determine the direction and amplitude of soil N transformation (Chen et al., 2017). Hence, we comprehensively analyzed the genes involved in the grid pathway of N-cycling (**Figure 2**). Taken together, the frequency and abundance of genes involved in glutamate synthesis were significantly higher than other N-cycle processes, while the abundance of genes involved in nitrification were the lowest (**Figure 2C**). This finding was similar to the result of Nelson’s metagenomic sequencing (Nelson et al., 2015), indicating that tea soils with different acidity in this study have the same characteristics of N metabolism pathways. However, the abundances of genes involved in N fixation and anammox were below the detection line and not captured (**Figure 2B**). This may be due to mineral forms of N that were readily available in the environment, requiring no or less metabolic investment according to the principle of resource acquisition (Zheng et al., 2017).

Compared with NO_3_^−^, NH_4_^+^ can more effectively promote the biosynthesis of free amino acids and catechins in tea leaves and roots, and significantly improve the activity of glutamine synthase, thus improve the tea quality (Huang et al., 2018; Wang et al., 2021). Our results found that the abundance of genes involved in glutamate synthesis in acidified soil decreased significantly, while it was positively correlated with NH_4_^+^-N (**Figures 2C**, **5**). This suggested that the content of NH_4_^+^-N in acidified soil decreased, which inhibited the synthesis of glutamate. However, it was not conducive to the accumulation and absorption of soil N content, thus reducing the N fertilizer use efficiency (Wang et al., 2020). The presence of NH_4_^+^ will improve the absorption rate of NO_3_^-^ (Ruan et al., 2016). Plants or microorganisms inhale NO_3_^−^-N into the body, reduce it to NH_4_^+^-N by assimilatory N reduction, and then synthesize N-related organic substances. And then the abundances of genes related to assimilatory N reduction pathway in ST were significantly higher than that in acidified soil (**Figure 2C**).

Our result was consistent with a previous study that reported that nitrification was negatively correlated with soil pH value (Jin et al., 2012). And high potential nitrification rates lead to low NH_4_^+^-N and to high NO_3_^−^-N (Jin et al., 2012; Li et al., 2018). Hence, the soil NO_3_^−^-N was significantly higher and NH_4_^+^-N was less after soil acidification (**Table 1**). Ammonification is a process in which microorganisms decompose organic matter and release NH_4_^+^ or NH_3_. Soil acidification promotes soil ammonification (**Figure 2C**), this may result in higher urease activity in acidified soil. But the content of NH_4_^+^-N in the acidified tea soil decreased, this suggested that the NH_4_^+^-N generated was partly absorbed by tea trees and partly converted to NO_3_^–^–N due to high nitrification, which also increased the risk of solidification or leaching, thus resulting in less NH_4_^+^-N in acidified soil. The results also indicated that the abundances of genes involved in denitrification increased significantly in acidified soil, and were negatively correlated with soil NH_4_^+^-N and available N, while were positively correlated with NO_3_^–^–N (**Figures 2C**, **5**). This was consistent with the results of Sun et al. (Sun et al., 2021). The contribution of denitrification to N_2_O production was significantly increased with the decrease of soil pH (Saggar et al., 2013; Wu et al., 2017). The transformation of N in soil was a cyclic process. The nitrification and denitrification of tea soil were enhanced after soil acidification, thus resulting in less NH_4_^+^-N in soil, which further inhibited glutamate synthesis (**Figure 5**).

Previous studies on genes related to soil N-cycling mainly focused on the relative abundance of individual genes involved in soil nitrification, denitrification and N-fixation, such as *nifH*, *amoA*, *nirS* and *nosZ* (Sun et al., 2021). However, other genes involved in N-cycling pathways have received less attention. Hence, we further comprehensively evaluated the abundance of key genes involved in each N-cycling processes. Among the glutamate synthesis-related genes, the abundances of *gltB* and *gltD* genes in tea soil with different acidity were consistent with the change trend of glutamate synthesis pathway (**Figures 2C**, **3A**), indicating that *gltB* and *gltD* play important roles in glutamate synthesis pathway. The changes in gene abundances in different individuals involved in the same pathway after acidification were not completely consistent (**Figure 3**), which suggested that the importance of studying the complete gene family involved in N-cycling process. Among the sequential nitrifying genes, we observed that the abundances of *amoA*/B/C in acidified soil were significantly increased. Soil nitrification begins with the oxidation of ammonia to nitrite, which is dominated by ammonia-oxidizing bacteria (AOB) and ammonia-oxidizing archaea (AOA) (Harter et al., 2014). The key control enzyme of this step is encoded by the *amoA* gene (Afzal et al., 2019). Yang et al. (2018a) reported that N fertilization stimulated nitrifying bacteria (*amoA*). Soil denitrification involves multiple N forms and functional genes (Lin et al., 2017). The denitrification process is mainly encoded by functional genes such as *narG* (NO_3_^-^ to NO_2_^-^), *nirS*/*nirK* (NO_2_^-^ to NO), *norB* (NO to N_2_O) and *nosZ* (N_2_O to N_2_) (Grassmann et al., 2022). The decrease of soil pH will reduce the availability of soil mineral N and organic carbon, and then indirectly affect N_2_O emission (Baggs et al., 2010). Several studies have shown that *narG* and *norB* genes significantly promoted N_2_O emission in acidic tropical forest soil (Tian et al., 2019). After acidification, the abundance of all genes involved in denitrification increased significantly (**Figure 3F**), which increases the risk of N_2_O emission.

### Soil microbial community composition and correlation analysis

Soil properties could indirectly mediate the N transformation process by affecting soil microbial community structure (Dong et al., 2018). In this research, significant differences were detected in soil N-related microbial community with AT and ST (**Figure S2**), which implied that soil acidification affected the soil N-related microbial community of tea plantation. Previous studies reported that Proteobacteria, Actinobacteria and Acidobacteria are the predominant microbial community phylum in terrestrial soil ecosystems (Liu et al., 2016; Delgado-Baquerizo et al., 2018). These results were consistent with ours, though the object of our study is the N-related microorganisms (**Figure 4**). Proteobacteria contains a large number of plant and animal pathogenic bacteria (Wagg et al., 2018). Previous study indicated that the abundance of Proteobacteria in diseased soil was relatively higher (Xue et al., 2015). Zhao et al. (2019) showed that a few parasitic actinomycetes can cause diseases of some plants and animals. Proteobacteria and Actinobacteria were increased after soil acidification (**Figure 4A**). This suggested that the content of pathogenic bacteria in tea soil increases after acidification. We also found that Chloroflexi, Nitrospirae and Firmicutes were significantly enriched in acidified soil (**Figure 4**). Several studies have shown that Chloroflexi and Nitrospirae and Firmicutes play nitrification or denitrification function in soil N-cycle (Kartal et al., 2007; Spieck et al., 2020). However, the abundances of Acidobacteria, Armatimonadetes, Verrucomicrobia and Thaumarchaeota in acidified soil decreased significantly (**Figure 4A**). We suggested that acidification could provide a bad survival environment for some soil beneficial microorganisms. Furthermore, we found that *Nitrospira*, *Rhodoblastus*, *Actinomadura*, *Thermogemmatispora* and *Mycobacterium* were significantly enriched in acidified soil (**Figure 4B**). Our result was consistent with a previous study that the abundance of *Nitrospir*a displayed a significant positive correlation with nitrification potential, and was negatively correlated with soil pH and high ammonia (Hu et al., 2021).

Many documents reported that soil microbial community composition is influenced by physicochemical factors (Zhang et al., 2022). RDA results indicated that some key rhizosphere microorganisms (Proteobacteria, Actinobacteria, Chloroflexi, Nitrospirae, Planctomycetes, Firmicutes, *Actinomadura*, *Nitrobacter*) had a positive correlation with soil NO_3_^–^–N, and had a negative correlation with pH, NH_4_^+^-N and available N (**Figure 6**). These results indicated that pH and soil N level were important factors driving soil microbial diversity and community structure change, which were in agreement with previous reports (Zhou et al., 2018; Li et al., 2020). Results also indicated that the beneficial microorganism in acidified soil decreased and the abundances of microorganisms involved in nitrification and denitrification increased, this increases the risk of soil N_2_O emission (Huang et al., 2015b).

## Conclusion

**Figure 7:**
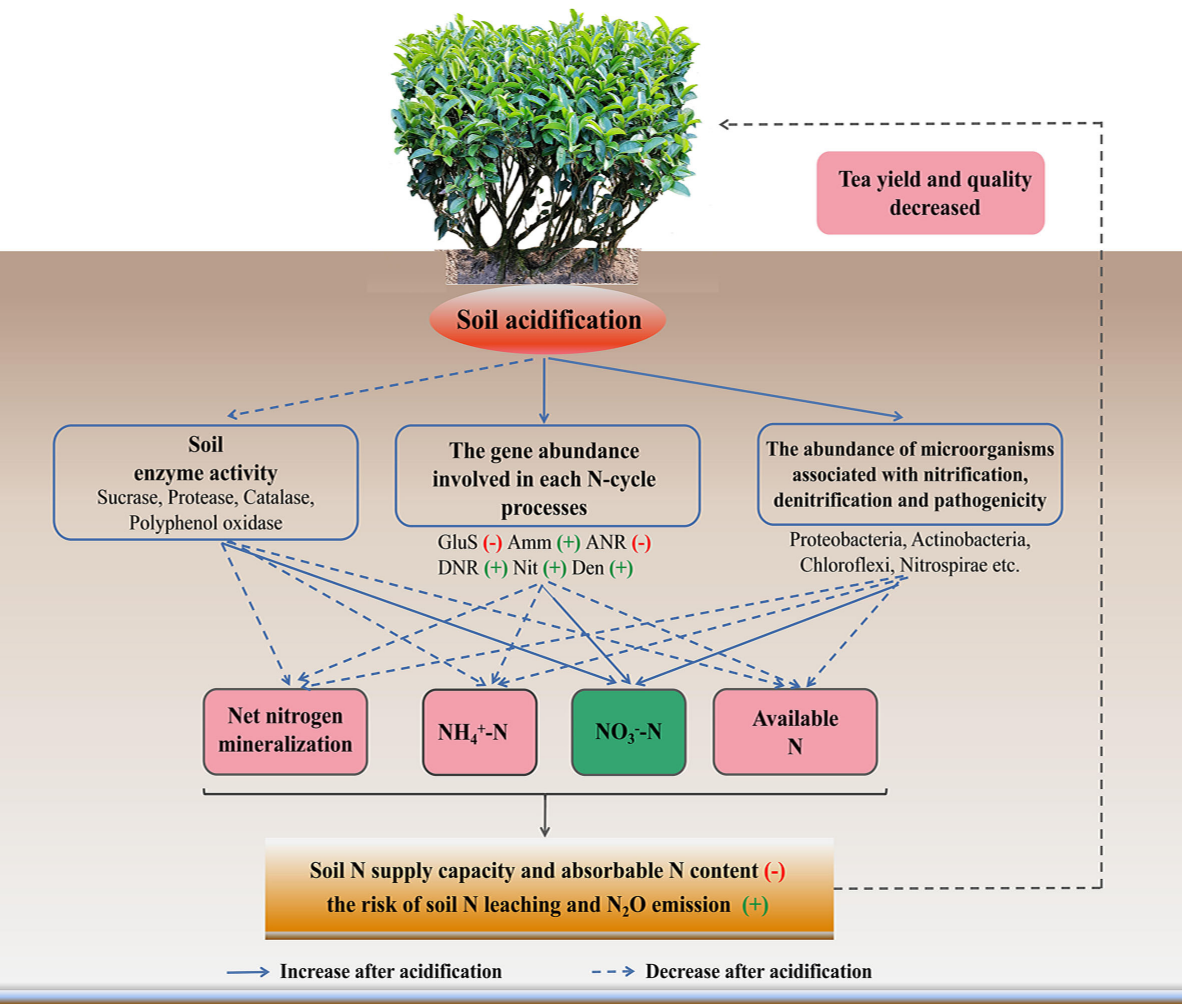
Model showing the mechanism of soil acidification on soil nitrogen form and transformation in tea gardens. The blue dotted and solid lines represent a decrease and increase in an indicator, respectively. Red minus sign indicates upregulated, while green plus sign indicates downregulated. GluS, glutamate synthesis; Amm, ammonification; ANR, assimilatory nitrogen reduction; DNR, dissimilatory nitrogen reduction; Nit, nitrification; Den, denitrification.

In conclusion, soil pH value affected the N cycling (**Figure 7**). After soil acidification, the soil enzyme activities involved in nutrient cycling decreased; the abundances of genes involved in glutamate synthesis and assimilatory N reduction increased significantly. Moreover, the abundances of genes involved in dissimilation N reduction, nitrification and denitrification increased significantly in acidified soil, and then affected the participation in the N-cycle of the microbial community composition, especially increased the amounts of pathogenic microorganism, and microorganisms related to nitrification and denitrification. These factors eventually lead to a decrease in the N supply capacity and the content of NH_4_^+^-N in tea soil, thus inhibited the growth of tea trees. In the future, a more comprehensive study is required to explore the response of microorganisms involved in each N transformation process respond to soil acidification in tea plantation.

## Data availability statement

The original contributions presented in the study are publicly available. This data can be found here: NCBI, PRJNA867479.

## Author contributions

SL designed and performed the experiments, analyzed the data and writing-original draft. ZL and YW investigation and sampling collection, performed the experiments. JL, GW and JY analyzed the data. HW conceived the experiments, approved the final manuscript. HH supervision, reviewed drafts and approved the final draft. All authors contributed to the article and approved the submitted version.

## Funding

This work was supported by National Natural Science Foundation of China (81973412), Natural Science Foundation of Fujian Province (2020J01369, 2020J01408), Project of Scientific Research of Young and Middle-aged Teachers, Fujian Province (JAT170573, JAT190761), Fujian Outstanding Research Talent Cultivation Project and Top Talent Training Program of Longyan University (2019JZ19).

## Conflict of interest

The authors declare that the research was conducted in the absence of any commercial or financial relationships that could be construed as a potential conflict of interest.

## Publisher’s note

All claims expressed in this article are solely those of the authors and do not necessarily represent those of their affiliated organizations, or those of the publisher, the editors and the reviewers. Any product that may be evaluated in this article, or claim that may be made by its manufacturer, is not guaranteed or endorsed by the publisher.
