# Supplementary material for: Soil metagenomic analysis on changes of functional genes and microorganisms involved in nitrogen-cycle processes of acidified tea soils
Source: Front Plant Sci. 2022 Oct 14;13:998178. doi: 10.3389/fpls.2022.998178 (PMC9614370; doi:10.3389/fpls.2022.998178)
Supplement: Supplementary file 1 [file DataSheet_1.docx]

Supplemental materials for

**Soil Metagenomic Analysis on Changes of Functional Genes and Microorganisms Involved in Nitrogen-cycle Processes of Acidified Tea Soils**

Shunxian Lin^1^, Zhijun Liu^1^, Yuchao Wang^1^, Jiayu Li^1^, Gege Wang^1^, Jianghua Ye^1,3^, Haibin Wang^*1,2^, Haibin He^*1^

1 Key Laboratory of Agroecological Processing and Safety Monitoring of Fujian Province, Fujian Agriculture and Forestry University, Fuzhou 35002, China.

2 College of Life Sciences, Longyan University, Longyan 364012, China.

3 College of Tea and Food Science, Wuyi University, Wuyishan 353400, China.

* Corresponding author:

Prof. Haibin He

College of Life Sciences, Fujian Agriculture and Forestry University;

Fuzhou 350002, Fujian, China;

1. mail: [alexhhb@163.com](mailto:alexhhb@163.com)

Prof. Haibin Wang

College of Life Sciences, Longyan University,

Longyan 364012, Fujian, China;

E-mail: [w13599084845@sina.com](mailto:w13599084845@sina.com)

**Supporting Information containing 5 pages, with Supplementary Method, 4 Tables and 3 Figure.**

**Supplementary Method**

***Detailed*** ***calculation of*** ***net N mineralization rates***

Net N mineralization rates were calculated using equation (1), (2), (3)

*Y_2_* = $\frac{\left( {NO}_{3}^{-}-N \right){}_{t}-\left( {NO}_{3}^{-}-N \right){}_{0}}{t}$ (1),

*Y_1_* = $\frac{\left( {NH}_{4}^{+}-N \right){}_{t}-\left( {NH}_{4}^{+}-N \right){}_{0}}{t}$ (2),

*Y_3_* = $\frac{\left( available N \right){}_{t}-\left( available \right){}_{0}}{t}$ (3),

Where

*Y_1_* is the nitrification rate;

*Y_2_* is the ammonification rate;

*Y_3_* is the nitrogen mineralization rate;

*(NH_4_^+^-N)_t_* and *(NH_4_^+^-N)_0_*, the NH_4_^+^-N contents before and after incubation, respectively;

*(NO_3_^-^-N)_t_* and *(NO_3_^-^-N)_0_*, the NO_3_^-^-N contents before and after incubation, respectively;

*(available)_t_* and *(available)_0_*, the available contents before and after incubation, respectively;

*t*, the days of cultivation.

**TABLE S1 Overview of the soil metagenomic sequencing**

| Sample | AT1 | AT2 | AT3 | ST1 | ST2 | ST3 | All/  Average |
| --- | --- | --- | --- | --- | --- | --- | --- |
| Raw base (Mbp) | 12306.03 | 13638.8 | 13277.64 | 12673.96 | 12248.1 | 12575.65 | 76720.18 |
| Clean base (Mbp) | 12272.37 | 13589.33 | 13228.89 | 12649.93 | 12237.05 | 12554.94 | 76532.51 |
| Effective rate (%) | 99.73 | 99.64 | 99.63 | 99.81 | 99.91 | 99.84 | 99.76 |
| Contigs | 596602 | 646239 | 633824 | 587283 | 601139 | 565114 | 605034 |
| Contigs bases (bp) | 568080528 | 636803691 | 607478406 | 627005627 | 608997201 | 566572171 | 602489604 |
| N50 (bp) | 955 | 991 | 962 | 1093 | 1016 | 996 | 1002 |
| N90 (bp) | 558 | 560 | 559 | 568 | 564 | 561 | 562 |
| Max (bp) | 149007 | 148989 | 75106 | 295799 | 293863 | 291927 | 209115 |
| Min (bp) | 500 | 500 | 500 | 500 | 500 | 500 | 500 |

AT, tea soil with a pH of 3.75; ST, tea soil with a pH of 5.26.

**TABLE S2 The detected gene probes number involving in N cycling**

| Gene category | No. of total gene probes detected | No. of gene probes detected in different soil samples | |
| --- | --- | --- | --- |
|  |  | AT | ST |
| Nitrogen cycling | 11049 | 9526 | 8764 |
| Glutamate synthesis | 4709 | 4114 | 3895 |
| Ammoniation | 1712 | 1496 | 1343 |
| Assimilatory nitrate reduction | 920 | 628 | 616 |
| Dissimilatory nitrate reduction | 1597 | 1374 | 1287 |
| Nitrification | 45 | 41 | 33 |
| Denitrification | 2066 | 1873 | 1590 |

AT, tea soil with a pH of 3.75; ST, tea soil with a pH of 5.26.

**TABLE S3 Relative abundances of the nitrogen-related total microbial top 10 genus in AT and ST**

| Phylum | AT | ST |
| --- | --- | --- |
| Proteobacteria | 44.25a | 42.63b |
| Actinobacteria | 20.68a | 17.19b |
| Acidobacteria | 8.39b | 15.08a |
| Chloroflexi | 4.45a | 3.98b |
| Nitrospirae | 5.97a | 0.85b |
| Planctomycetes | 2.08a | 2.02a |
| Firmicutes | 1.29a | 0.97b |
| Armatimonadetes | 0.86b | 1.07a |
| Verrucomicrobia | 0.49b | 1.12a |
| Thaumarchaeota | 0.40b | 1.07a |
| Others | 11.16b | 14.00a |

AT, tea soil with a pH of 3.75; ST, tea soil with a pH of 5.26.

**TABLE S4 Relative abundances of the nitrogen-related total microbial top 10 genus in AT and ST**

| phylum | genera | AT | ST |
| --- | --- | --- | --- |
| Proteobacteria | *Bradyrhizobium* | 3.189a | 3.129a |
| Nitrospirae | *Nitrospira* | 4.674a | 0.654b |
| Proteobacteria | *Rhodanobacter* | 4.058a | 1.270b |
| Actinobacteria | *Actinomadura* | 2.624a | 1.774b |
| Chloroflexi | *Thermogemmatispora* | 1.747a | 1.189b |
| Acidobacteria | *Candidatus Solibacter* | 0.952b | 2.018a |
| Proteobacteria | *Geobacter* | 1.023b | 1.473a |
| Actinobacteria | *Mycobacterium* | 1.319a | 0.914b |
| Chloroflexi | *Ktedonobacter* | 0.937b | 1.032a |
| Proteobacteria | *Nitrobacter* | 0.730b | 1.168a |
|  | Others | 78.748b | 85.379a |

AT, tea soil with a pH of 3.75; ST, tea soil with a pH of 5.26.

**FIGURES**

|  |  |
| --- | --- |

**FIGURE S1 Total detection frequency (A) and gene abundance (B) related to nitrogen cycling in tea soil with different pH values.** Different lowercases represent significant differences at p < 0.05 level.


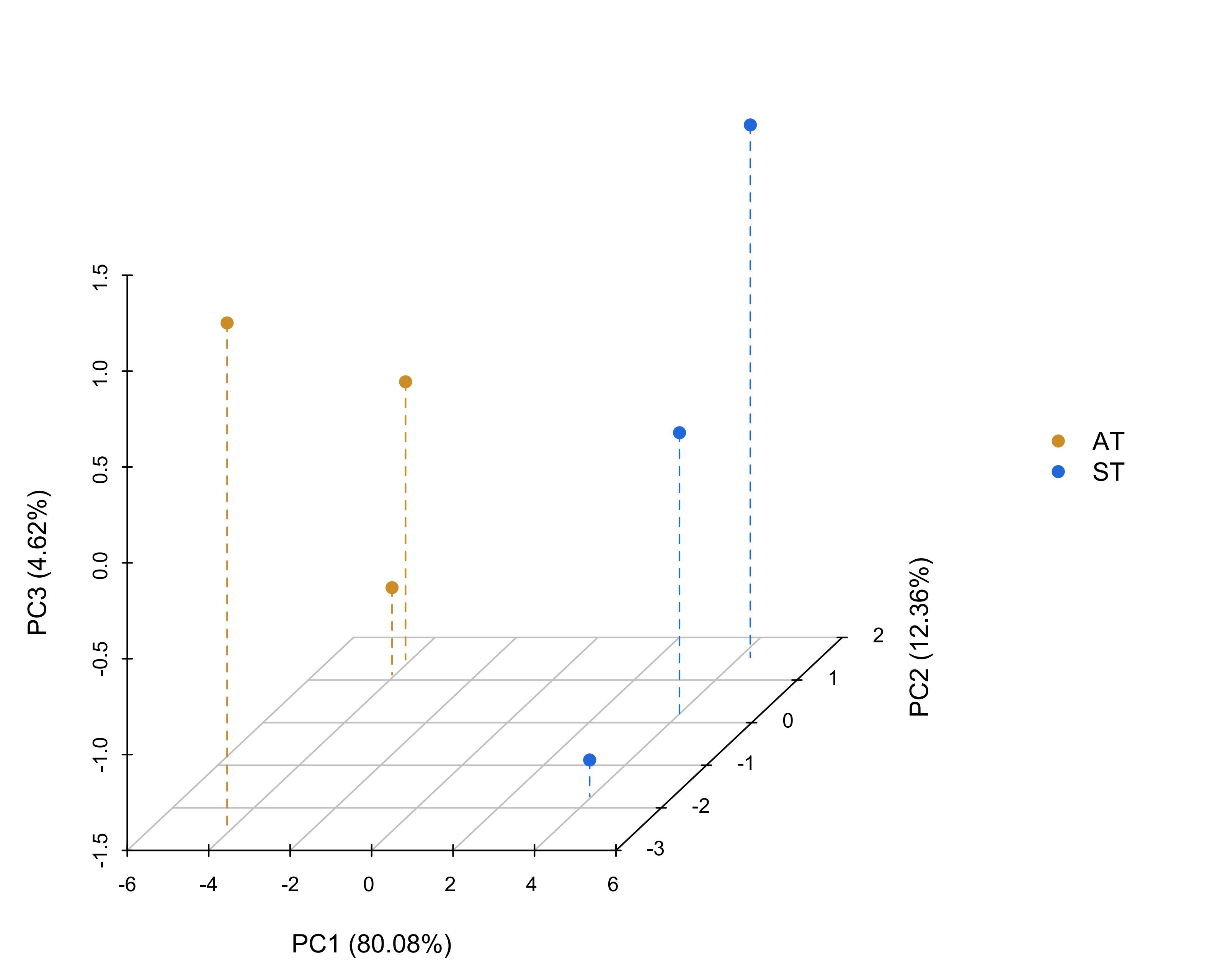


**FIGURE S2 PCA analysis of nitrogen-related total microorganism with different pH value of tea soils.**


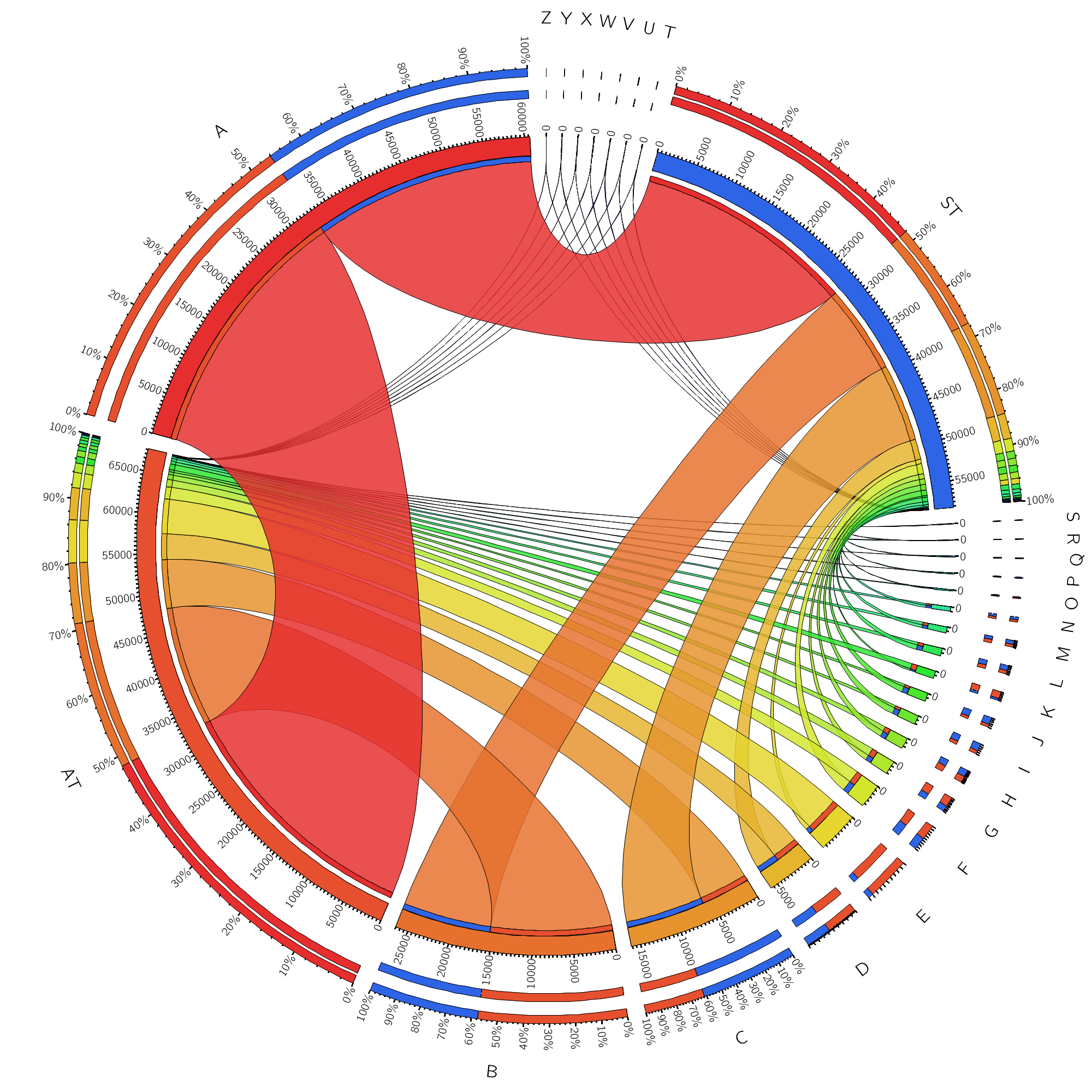


**FIGURE S3 Distribution of** **nitrogen-related total microbial phyla with different pH value of tea soils.**

AT, tea soil with a pH of 3.75; ST, tea soil with a pH of 5.26. A, Proteobacteria; B, Actinobacteria; C, Acidobacteria; D, Chloroflexi; E, Nitrospirae; F, Planctomycetes; G, Firmicutes; H, Armatimonadetes; I, Verrucomicrobia; J, Thaumarchaeota; K, Bacteroidetes; L, Euryarchaeota; M, Gemmatimonadetes; N, Candidatus Rokubacteria; O, Candidatus Omnitrophica; P, Cyanobacteria; Q, Candidatus Kryptonia; R, Crenarchaeota; S, Elusimicrobia; T, Euryarchaeota; U, Candidatus Handelsmanbacteria; V, Candidate division NC10; W, Candidatus Lindowbacteria; X, Candidatus Bathyarchaeota; Y, Candidatus Tectomicrobia; Z, Spirochaetes.
